# SH3 Domains Differentially Stimulate Distinct Dynamin I Assembly Modes and G Domain Activity

**DOI:** 10.1371/journal.pone.0144609

**Published:** 2015-12-10

**Authors:** Sai Krishnan, Michael Collett, Phillip J. Robinson

**Affiliations:** Cell Signalling Unit, Children’s Medical Research Institute, The University of Sydney, Westmead, New South Wales, Australia; Hungarian Academy of Sciences, HUNGARY

## Abstract

Dynamin I is a highly regulated GTPase enzyme enriched in nerve terminals which mediates vesicle fission during synaptic vesicle endocytosis. One regulatory mechanism involves its interactions with proteins containing Src homology 3 (SH3) domains. At least 30 SH3 domain-containing proteins bind dynamin at its proline-rich domain (PRD). Those that stimulate dynamin activity act by promoting its oligomerisation. We undertook a systematic parallel screening of 13 glutathione-*S*-transferase (GST)-tagged endocytosis-related SH3 domains on dynamin binding, GTPase activity and oligomerisation. No correlation was found between dynamin binding and their potency to stimulate GTPase activity. There was limited correlation between the extent of their ability to stimulate dynamin activity and the level of oligomerisation, indicating an as yet uncharacterised allosteric coupling of the PRD and G domain. We examined the two variants, dynamin Iab and Ibb, which differ in the alternately splice middle domain α2 helix. They responded differently to the panel of SH3s, with the extent of stimulation between the splice variants varying greatly between the SH3s. This study reveals that SH3 binding can act as a heterotropic allosteric regulator of the G domain via the middle domain α2 helix, suggesting an involvement of this helix in communicating the PRD-mediated allostery. This indicates that SH3 binding both stabilises multiple conformations of the tetrameric building block of dynamin, and promotes assembly of dynamin-SH3 complexes with distinct rates of GTP hydrolysis.

## Introduction

The ‘classical’ dynamins are a three gene family comprising dynamin I, II and III involved in clathrin-mediated endocytosis (CME). They are highly conserved 94 kDa GTPases with 70% amino acid sequence identity [1]. They are multidomain proteins consisting of: a GTPase (G) domain, bundle signalling element (BSE), middle domain, the lipid binding pleckstrin homology (PH) domain, and a proline-rich domain (PRD) which is the binding site of SH3 domains [2]. Dynamin I is primarily expressed in neurons and is enriched in nerve terminals, where it has a specialised function in the recycling of synaptic vesicles, via synaptic vesicle endocytosis (SVE) [3].

Dynamin oligomerises into higher order assembly states, depending on the experimental conditions: tetramer, ring and helices. Its GTPase activity is relatively low in the tetrameric state, and greatly increases with oligomerisation, with the dynamin helix being the most active dynamin complex [4,5]. Other members of the larger dynamin-like family are also stimulated by oligomerisation, since assembly-deficient mutants inhibit GTPase activity across the family [6–8]. It is likely that GTP hydrolysis signals structural changes back to the PH domain to allow it to complete lipid fission [9], suggesting the existence of allosteric networks within dynamin capable of conveying these signals. Dynamin primarily exists as a tetramer in solution [10–12], built on a dimer-of-dimers [13]. Dynamin helices consist of thousands of monomers and require an assembly-template to be formed *in vitro*, such as liposomes, microtubules or nanotubes [5,14,15]. Helices have been associated with endocytosis. Dynamin rings are approximately 28-mer soluble complexes that form through self-assembly *in vitro* at high dynamin concentrations [4,16,17], at lower concentrations when in the GTP-bound transition state [18], or by lowering the salt concentration below physiological levels [16]. Dynamin rings have been functionally associated with actin dynamics [19].

Specific SH3 domains also stimulate ring oligomerisation *in vitro*. The SH3 domain is one of the most abundant protein-protein binding modules in the mammalian genome with approximately 300 SH3 proteins [20,21]. SH3 domains range in size from 50–70 residues and bind specifically to proline-rich sequence motifs in a large array of target proteins [22–24]. There are ~30 dynamin-binding SH3 domains which have signalling, endocytic or actin regulatory functions [25]. Some of these SH3 domain proteins, such as grb2, SNX9, endophilin, intersectin, cortactin, are able to stimulate dynamin activity via oligomerisation [26–30]. This stimulation has been associated with the assembly of dynamin into oligomers, confirmed by obtaining sedimentable dynamin pellets and electron microscopy showing dynamin ring formation [28–30]. This supports the broad array of evidence that dynamin oligomerisation determines the activity of the G domain.

There has been no comprehensive study of the effects of isolated SH3 domains on the assembly and activity of dynamin. Most prior studies use the multidomain full length form of the protein, often including the dimerising BAR domain, obscuring the effect of the SH3 domain itself. Interpretation of previous studies is also complicated by inconsistent assay conditions and lack of parallel experimentation. Thus, it remains unclear which endocytosis- or actin-related SH3 domains regulate dynamin activity and to what relative extents. We have conducted the first systematic study of SH3 domain-stimulated dynamin activity and assembly using of a panel of 13 GST-SH3 domains, confirming that there is no correlation between the relative binding of these SH3s to dynamin I and its GTPase activity. While assembly-independent activity was not observed, only a limited relationship was found between the extent of dynamin activity and assembly in the presence of certain SH3 domains. This reveals that SH3 domains can induce multiple conformation states of dynamin with specific hydrolysis rates. Since oligomerisation requires the middle domain, we capitalised on naturally occurring splice variants of its α2 helix and demonstrated that the middle domain plays a role in communicating allosteric signals from the PRD to the G domain.

## Material and Methods

### Constructs

A panel of 13 SH3 domain constructs were used to test their effect on dynamin. They were chosen as those SH3 domains known to bind dynamin and which also have a role in endocytosis. Human amphiphysin I SH3, human Formin Binding Protein 17 SH3 (FBP17) and full length human Cdc42 interacting protein 4 (CIP4) in pGEX-6P-1 vector were originally provided by Pietro de Camilli (Yale University School of Medicine, USA). Human amphiphysin II SH3 was in pGEX-2T vector and provided by Pietro de Camilli. Mouse endophilin I SH3 and *Xenopus leavis* intersectin I SH3 A/B/C/D/E were in pGEX-2T and were provided by Peter McPherson (McGill University, Canada). Mouse syndapin I SH3 was in pGEX-6P-1 and provided by Markus Plomann (University of Cologne, Germany). Human sorting-nexin 9 (SNX9) SH3 was in pGEX-6P-1 generated from SNX9 cDNA supplied by Sandra Schmid (The Scripps Research Institute, USA). Bovine p85 SH3 domain was in pGEX-2T and provided Tony Pawson (Mount Sinai Hospital, Toronto, Ontario, Canada). Full-length mouse Actin binding protein 1 (Abp1) and mouse cortactin were from www.addgene.org and were originally in the pEGFP plasmid construct. For all the full-length constructs the SH3 domain was amplified and subcloned into pGEX-6P-1 (Amersham biosciences).

### SH3 domain expression and quantification

All glutathione S-transferase (GST)-fusion SH3 proteins were transformed into *Escherichia coli* and cultured in LB medium supplemented with ampicillin. Protein expression was induced with 1 mM isopropyl β-D-1-thiogalactopyranoside (IPTG) for 4 hours at 37°C. The culture was harvested and sonicated with ice cold salt/Tris/EDTA (STE) buffer (300 mM NaCl, 10 mM Tris final pH 8.0 and 1 mM EDTA) containing 1% (v/v) Triton X-100, 1 mM DTT, 10 μg/ml leupeptin, 1 mM phenyl methyl sulfonyl fluoride and 1 EDTA free protease cocktail inhibitor tablet. Following centrifugation at 48,254 x g for 15 minutes, the supernatant containing the GST-SH3 protein was incubated with glutathione-sepharose beads (Amersham Biosciences, Piscataway, NJ, USA) according to the manufacturer’s instructions. GST-SH3 solution was obtained by eluting it from glutathione-sepharose beads using 10 mM reduced glutathione in 50 mM Tris-HCl, pH 8.0 and overnight dialysis.

High sensitivity amino acid analysis was used for protein quantification of the GST-intersectin I SH3E by gas phase hydrolysis (Australian Proteome Analysis Facility). It was then used was used as the reference protein against which the other 12 recombinant GST-SH3 domains and dynamin were normalised with by SDS-acrylamide gels and Coomassie Blue staining.

### Dynamin purification

Recombinant human dynamin I middle domain splice variants Iab and Ibb (splice nomenclature is from [31]) were expressed as full-length proteins, containing an N-terminal six his-tag, in Sf21 insect cells. The spliced tail was consistently the short b variant in this study. The specific splice variants and endogenous sheep brain dynamin I were all purified as described previously [32].

### Kinetic GTPase assays

Dynamin activity was analysed using an Enzyme Linked Inorganic Phosphate Assay (ELIPA) modified from the ELIPA ATPase assay kit (Cytoskeleton Inc., CO) [33]. All assays were performed with dynamin I (100 nM) and GST-SH3 (40 μg/ml for all 13) pre-incubated for 60 minutes in reaction buffer containing 10 mM Tris pH 7.4, 30 mM or 150 mM NaCl, 20 mM MgCl_2_, 0.05% v/v Tween 80, 1 μg/ml leupeptin and 0.1 μM AEBSF. This was followed by the addition of 300 μM GTP, 160 μM AEBSF and 1 U/ml purine nucleoside phosphorylase to initiate the reaction. The absorbance was read at 360 nm every 30 seconds for duration of 1 hour using the VersaMax^™^ micro-plate reader (Molecular Devices, USA).

The GTPase rates (μg Pi/mg/min) were determined by linear regression analysis on the initial 2–30 minutes of the time course with a longer interval of up to 60 minutes being used for assays that showed lower activity. The increase in activity during this period was linear, with the coefficient of determination (R^2^) typically greater than 0.95. The basal rate of dynamin activity (dynamin alone, without SH3 domain stimulation) was calculated separately and was subtracted from the respective values, so that only the GST-SH3 stimulated effects are shown in this study.

### Dynamin assembly

Dynamin assembly was assessed by collecting oligomerised dynamin trapped on paper filter spin cups (from Thermofisher Scientific (IL, USA)) in a form ready to run on SDS-acrylamide gels without further concentration or precipitation. Dynamin I (100 nM / 9.6 μg/ml) was preassembled in the presence of SH3 domains (40 μg/ml) in reaction buffer containing 10 mM Tris pH 7.4, 30 mM NaCl, 20 mM MgCl_2_, 0.05% v/v Tween 80, 1 μg/ml leupeptin and 0.1 μM AEBSF. The preassembly was performed for 60 minutes at 37°C with shaking at 700 RPM followed by addition of 300 μM GTP and further incubation for the same duration. The reaction mix was transferred from the microfuge tube to the spin cups which had been previously washed three times with 1% Triton X-100. The samples were centrifuged at room temperature for 2 minutes at 18,000 x g and the supernatants were collected and diluted with SDS-PAGE sample buffer followed by heating at 85°C. The reaction volume (150 μl) for assembly assay was the same as for the GTPase assay, to ensure enough dynamin could be visualised on the gel. Oligomerised dynamin was eluted with 30 μl of 2X SDS-PAGE sample buffer, pre-heated to 85°C, and the spin cup was then centrifuged at room temperature for 5 minutes at 18,000 x g. The ‘pellet’ (paper-trapped) and supernatant samples are resolved on a 20 cm long 10% SDS-acrylamide gels and stained with colloidal Coomassie Blue. Assembly was quantified using the ImageQuant TL (GE Healthcare) software after densitometric analysis of the pellet (scanned with ImageScanner III LabScan 6.0 (GE Healthcare)) and is presented as a fold change relative to the basal (‘No SH3’) control.

### Pull-down experiments

Equal amounts of various GST-SH3 domain proteins still bound on GSH sepharose beads were incubated with purified dynamin I or synaptosome lysate. The incubation of purified dynamin (~3 μg) with GST-SH3 proteins was done in buffer containing 10 mM Tris pH 7.4, 30 mM or 150 mM NaCl, 20 mM MgCl_2_, 0.05% v/v Tween 80, 1 μg/ml leupeptin, 1 mM EDTA and 1 mM EGTA.

Synaptosomes (the P2 pellet) were freshly isolated nerve terminals from rat brain tissue as described previously [34]. Synaptosomes predominantly expresses the dynamin I isoform by about 50-fold over dynamin II and III [35,36]. They were lysed in a buffer containing 1% (v/v) Triton X-100, 1 mM EDTA, 1 mM EGTA, 5 mM Tris pH 7.4, 30 mM NaCl, 10 μg/ml leupeptin, 1 mM PMSF and 1 EDTA-free protease inhibitor tablet/20 ml. The homogenate was immediately centrifuged, at 48,384 x g for 15 minutes at 4°C to collect the supernatant which is the synaptosomal lysate.

The incubation was performed in a Mini BioSpin^™^ chromatography column (Bio-Rad Lab (CA, USA)) with rotation for 10 minutes at 4°C for the purified sheep dynamin I, or 30 minutes for the human dynamin I splice variants. The beads were washed extensively using PBS. Proteins were eluted in 2X SDS-PAGE sample buffers with heating at 85°C, followed by centrifugation to collect the solubilised proteins [37]. Dynamin binding was visualised by resolving the samples on 20 cm long 10% SDS-acrylamide gels and staining with colloidal Coomassie Blue.

### Ethics Statement

The isolation of organs from animals was done with approval from the Animal Care and Ethics Committee for the Children's Medical Research Institute, Sydney, Australia.

## Results

### Regulation of the dynamin G domain by SH3 domains is independent of the extent of binding or oligomerisation

**Fig 1:**
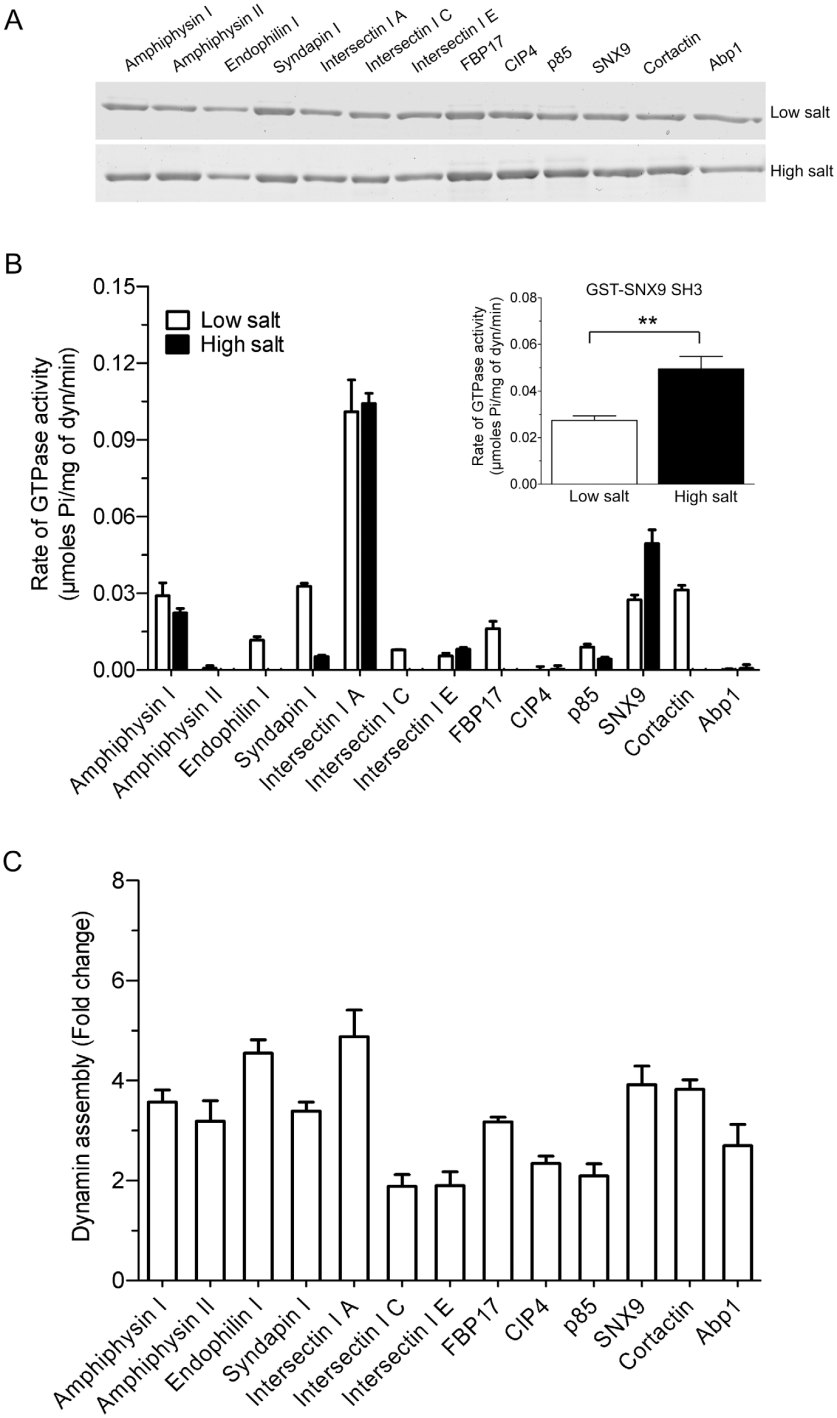
Lack of correlation between SH3 binding, stimulation and assembly of dynamin. **(A)** The relative binding of dynamin to individual GST-SH3 domains (~12 μg) was compared by performing pull-down analysis using purified sheep dynamin I (~3 μg) in the presence of low or high salt. The pull-down was resolved on 10% SDS-acrylamide gels and a Coomassie Blue stained gel is shown. The image are representative of n = 3 independent experiments. **(B)** The effect of purified recombinant GST-SH3 domains (40 μg/ml) on stimulation of purified sheep brain dynamin I (100 nM / 10 μg/ml) GTPase activity using ELIPA in the presence of low (30 mM NaCl) or high (150 mM NaCl) salt. All data is mean ± S.E.M. and n ≥ 3. The inset shows a zoom of the low and high salt values for GST-SNX9 SH3. A two-tailed Student t-test showed significance (*, p < 0.05). **(C)** Relative ability of 13 GST-SH3 domains to oligomerise dynamin using a sedimentation assay. GST-SH3 domain stimulated dynamin I assembly obtained from densitometric analysis of the dynamin pellet (see S2 Fig). The data is representative of n = 3 independent experiments performed in duplicate and is expressed as fold change relative to dynamin basal (no SH3) control.

A panel of 13 bacterially expressed dynamin-binding GST-SH3 domains were immobilised on GSH-agarose beads and used in pull-down experiments to assess their relative binding to purified sheep brain dynamin I. The GST-SH3 domains bound dynamin to varying extents, with no discernible difference observed between the low and high salt conditions (Fig 1A). The binding pattern to purified dynamin I also broadly matched the data obtained using synaptosomal lysate, despite the presence of other potential binding proteins in the lysate (S1 Fig). Intersectin I SH3A was relatively weaker at dynamin binding yet elicited the highest stimulation under both ionic conditions. Syndapin I, FBP17 and CIP4 had no stimulatory effect under high salt and yet bound dynamin sufficiently well under this condition.

The effect of the GST-SH3 domains on stimulating dynamin GTPase activity was determined using an ELIPA GTPase assay to determine rates, rather than use of an end-point assay. The dynamin concentration used (100 nM / 10 μg/ml) was well below that required to elicit spontaneous self-assembly (>600 nM) [16,38,39]. Under ‘low salt’ assay conditions, each of the 13 GST-SH3 domains, except amphiphysin II and CIP4, stimulated dynamin activity (Fig 1B). Dynamin assembly *in vitro* is highly dependent on salt concentration, since increased ionic strength reduces assembly and thus activity [4,16]. Salt bridges are a key part of dynamin tetramer formation [2] and we aimed to find SH3 domains capable of stimulating dynamin in the presence of a ‘high salt’ (150 mM NaCl) buffer. High salt reduced or almost abolished the stimulation of dynamin activity for most GST-SH3s (Fig 1B). Surprisingly, high salt significantly increased the ability of GST-SNX9 SH3 to stimulate the rate of dynamin activity (Fig 1B inset). This is despite the observation that, under low salt, this SH3 has a relatively similar level of stimulation to amphiphysin I, syndapin I and cortactin GST-SH3 domains, which show reduced stimulation in high salt buffer. This indicates no relationship between the extent of dynamin binding and rate of stimulation by these endocytic SH3 domains, greatly extending previous observations [40].

We next compared the relative ability of all 13 GST-SH3 domains to oligomerise dynamin using a sedimentation assay under the same buffer conditions as the ‘low salt’ assay. In the absence of SH3 domains dynamin was found primarily in the supernatant, while the SH3 domains induced accumulation of dynamin into the ‘pellet’, indicating increased assembly (S2 Fig). Quantification of the pellet by densitometry showed that the extent of assembly varied greatly for each SH3 domain (Fig 1C). By expressing activity and assembly stimulation as a fold-change over dynamin in the absence of SH3, these two properties were compared by rank order analysis (Table 1). Most SH3 domains showed little discrepancy between the two variables in their ranking but there were some striking exceptions. Syndapin I was one of the best stimulators of dynamin GTPase activity but a relatively moderate promoter of its assembly with endophilin I showing the opposite trend. Amphiphysin II, Abp1 and CIP4 were barely able to stimulate dynamin activity above basal and yet achieved a significant increase in assembled dynamin. It was also notable that while intersectin I A was an outlier relative to the other SH3 domains in promoting dynamin activity, yet this did not occur in the assembly assay. The two biochemical properties of dynamin compared here were previously held to be directly linked, such that one has been used as predictor of another [4,5,16,41]. Our results show this relationship does not universally apply.

**Table 1:** Rank order analysis of the stimulation of sheep dynamin I activity (Fig 1B) and assembly (Fig 1C) by 13 GST-SH3 domains.

GST-SH3 domains	Activity (fold change)	GST-SH3 domains	Assembly (fold change)
Intersectin I A	14.68	Intersectin I A	4.88
**Syndapin I**	5.90	**Endophilin I**	4.55
Cortactin	5.49	SNX9	3.92
Amphiphysin I	5.32	Cortactin	3.83
SNX9	5.12	Amphiphysin I	3.57
FBP17	3.97	**Syndapin I**	3.39
**Endophilin I**	2.82	**Amphiphysin II**	**3.19**
p85	2.24	FBP17	3.17
Intersectin I C	2.10	**Abp1**	**2.69**
Intersectin I E	1.83	**CIP4**	**2.34**
**Amphiphysin II**	**1.09**	p85	2.09
**Abp1**	**1.03**	Intersectin I E	1.90
**CIP4**	**0.83**	Intersectin I C	1.88

The GST-SH3 domains are listed in descending order of their stimulatory effect on endogenous sheep brain dynamin I activity and assembly under low salt conditions. Both parameters are represented as fold change relative to dynamin basal (no SH3) control. Those exhibiting poor correlation between activity and assembly are indicated in bold.

Overall, these data show intersectin I SH3A was the best stimulator of dynamin, with SNX9 being a standout protein in its ability to better promote dynamin activity under high ionic conditions. The novel observation that dynamin assembly and activity can be disconnected suggests the possible existence of a GTPase stimulation mechanism in addition to assembly. Thus, each dynamin-SH3 complex has specific hydrolysis rates suggesting they may be mediated by each one producing a distinct dynamin oligomer conformation.

### The middle domain facilitates SH3 domain allosteric regulation of the G domain

**Fig 2:**
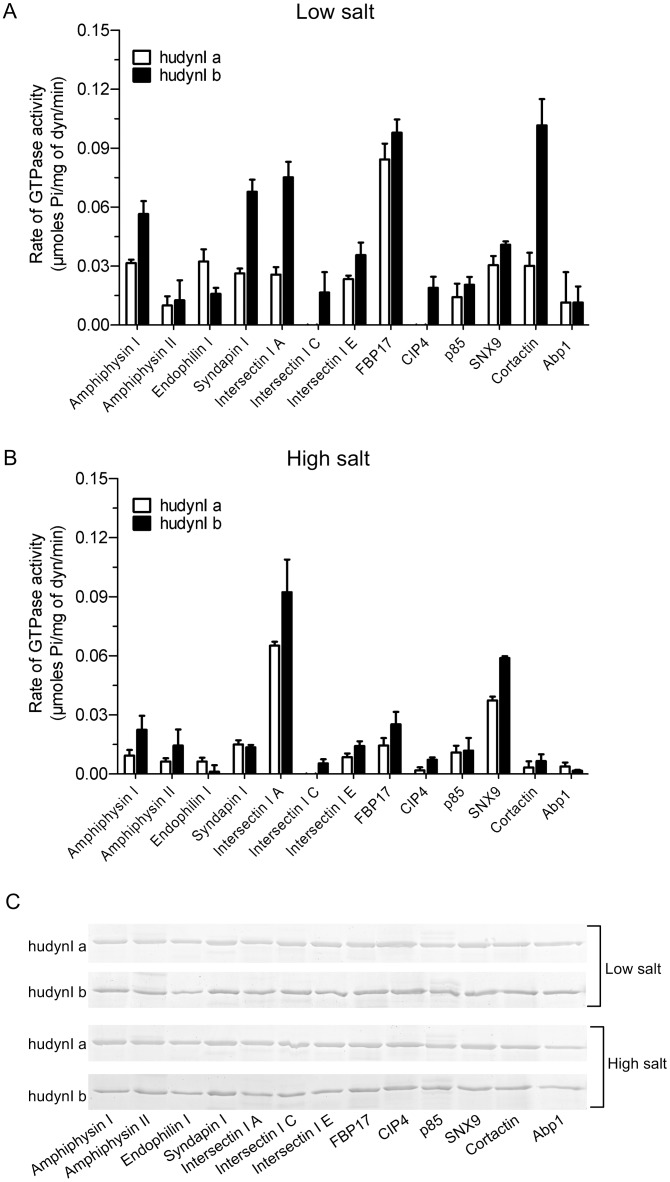
Stimulation of the dynamin GTPase activity by SH3 domains is determined by its middle domain. Effect of GST-SH3 domains (40 μg/ml) on the GTPase activity rate of purified recombinant human dynamin I middle domain splice variants ab and bb (hudynI a and b, both at 100 nM / 10 μg/ml) determined using ELIPA. The assays were performed in the presence of low salt (30 mM NaCl) **(A)** or high salt (150 mM NaCl) **(B)**. All data is shown as means ± S.E.M. and n ≥ 2. **(C)** The relative binding of dynamin to individual GST-SH3 domains (~12 μg) was compared by performing pull-down analysis using purified recombinant full-length hudynI a and b splice variants (~3 μg). The pull-down was performed in the presence of low or high salt for both splice variants. Dynamin was resolved on 10% SDS-acrylamide gels and a Coomassie Blue stained gel is shown. The images are representative of n = 2 independent experiments.

Since oligomerisation requires the middle domain of dynamin we capitalised on the occurrence of two naturally occurring dynamin I middle domain splice variants ‘a’ and ‘b’ that are highly sequence-conserved across mammalian species (S3 Fig). The spliced exon produces two versions of the α2 helix of the 45 amino acid middle domain 4-helical bundle. This α2 helix does not appear to be engaged in dimer formation, but lies at the interface between the two dimers in the dynamin tetramer structure [2]. Its unique location and preliminary mutational analysis suggests that the spliced α2 helix may play a key role in dynamin allosteric regulation between two dimer pairs or in oligomerisation of multiple tetramers, rather than within a monomer or dimer [13]. We therefore asked whether the α2 helix may be involved in allosteric regulation of the G domain via SH3-PRD interactions, by comparing activity of two human dynamin middle domain splice variants, Iab and Ibb (hereafter simplified to Ia and Ib to refer to middle domain splicing). Firstly, the GST-SH3 domains were each tested under low salt conditions (Fig 2A). All, except that of endophilin I, preferentially stimulated the activity of Ib. Strikingly, the SH3 domain of endophilin I showed the opposite effect, being more selective for stimulating Ia. The data suggests that different SH3 domains stabilize dynamin in different conformations mediated via the spliced α2 helix.

The activity data for the two human middle domain splice variants were then compared with that of the endogenous sheep brain dynamin I (Table 2), which comprises a mixture of splice variants each of which retains identical amino acid sequences for both middle domain α2 helices (S3 Fig). The stimulation by each SH3 domain was compared by rank order analysis to the low salt sheep dynamin I data (from Fig 1A). Notably, GST-FBP17 SH3 was a very potent stimulator of both Ia and Ib whereas it evoked 5 to 6-fold less activity from sheep dynamin I. GST-intersectin I SH3A stimulated brain dynamin I and dynamin Ib to about the same rate, yet was only moderately effective on Ia. Contrastingly, GST-endophilin I was around 2-fold more potent in stimulating Ia than either Ib or brain dynamin I. GST-cortactin SH3 was over 3-fold more potent on Ib variant than Ia or brain dynamin I. GST-SH3 domains of Abp1, CIP4 and intersectin I C were notable in being completely inactive on certain dynamins but not on others.

**Table 2:** Rank order analysis of the stimulation of the activity of endogenous sheep dynamin I (from Fig 1B) and single splice variants human dynamin Iab and Ibb (Fig 2A) by 13 GST-SH3 domains.

Sheep dynamin I		Human dynamin Ia		Human dynamin Ib	
GST-SH3 domain	Activity rate	GST-SH3 domain	Activity rate	GST-SH3 domain	Activity rate
**Intersectin I A**	**0.101**	**FBP17**	**0.084**	**Cortactin**	**0.102**
Syndapin I	0.033	**Endophilin I**	**0.032**	**FBP17**	**0.098**
**Cortactin**	**0.031**	Amphiphysin I	0.032	**Intersectin I A**	**0.075**
Amphiphysin I	0.029	SNX9	0.030	Syndapin I	0.068
SNX9	0.027	**Cortactin**	**0.030**	Amphiphysin I	0.056
**FBP17**	**0.016**	Syndapin I	0.026	SNX9	0.041
**Endophilin I**	**0.012**	**Intersectin I A**	**0.026**	Intersectin I E	0.036
p85	0.009	Intersectin I E	0.023	p85	0.020
**Intersectin I C**	**0.008**	p85	0.014	**CIP4**	**0.019**
Intersectin I E	0.005	**Abp1**	**0.011**	**Intersectin I C**	**0.017**
Amphiphysin II	0.001	Amphiphysin II	0.010	**Endophilin I**	**0.016**
**Abp1**	**0.000**	**Intersectin I C**	**0.000**	Amphiphysin II	0.013
**CIP4**	**0.000**	**CIP4**	**0.000**	**Abp1**	**0.011**

The rate of dynamin GTPase activity (μmoles Pi/mg of dyn/min) shown was generated under low salt assay conditions. The GST-SH3 domains are listed in descending order of their stimulatory effect across the three dynamins on sheep brain dynamin I. Those exhibiting poor correlation with sheep dynamin I, either in terms of stimulation or rank order, are indicated in bold.

Pull-down analysis showed the binding pattern of the SH3s to the two splice variants was similar to that observed for sheep dynamin I with no difference in binding between the salt conditions (Fig 2C). Yet again no correlation was observed between activity and binding. There was also no change in the binding pattern between the Ia and Ib middle domain isoforms, which was not surprising given that they both contain the same PRD sequence. This result highlights the important role of the middle domain in the allosteric regulation of GTPase activity since the two isoforms have variable stimulation despite no difference in the PRD-SH3 interaction between them. Therefore the SH3 domains have a remarkably varied effect on the activity of the two splice variants, despite them having the same PRD. This indicates markedly distinct allostery between the splice variants.

**Fig 3:**
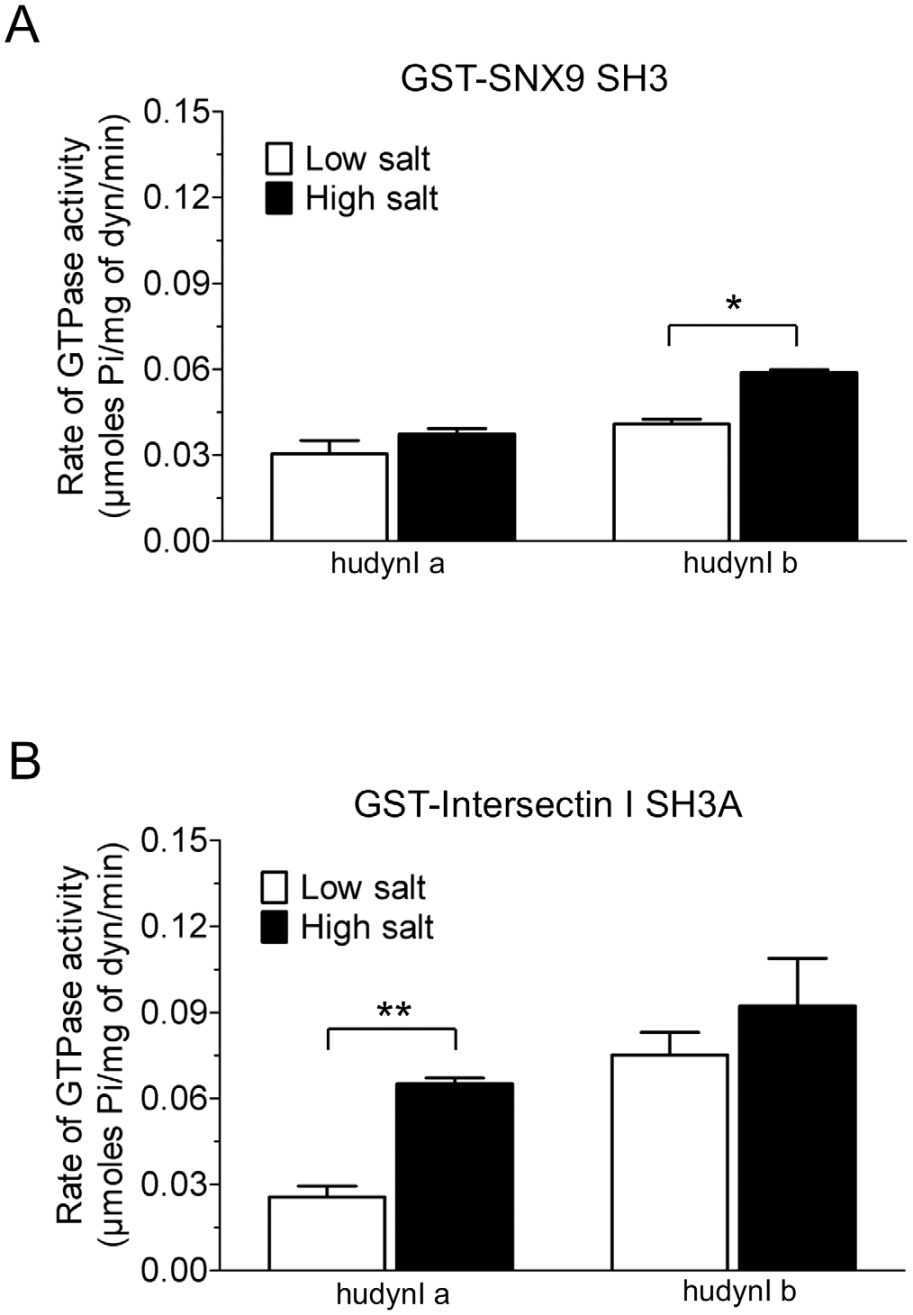
Effect of salt on the stimulation of dynamin I middle domain splice variants by GST-SNX9 SH3 and GST-intersectin I SH3A. Statistical analysis by two-way ANOVA followed by Bonferroni’s post-test shows GST-SNX9 SH3 **(A)** and GST-intersectin I SH3A (B) elicit a greater rate of GTPase activity in the presence of high salt for both splice variants. All data shown is ± S.E.M. with n ≥ 2 (*, p < 0.05 and **, p < 0.01).

In high salt buffer all GST-SH3s, with the exception of SNX9 and intersectin I A, elicited reduced activity of less than 0.03 μmoles Pi/mg of dynamin for both the splice variants, similar to the effect of salt on brain dynamin I (Fig 2B). However, some of the splice form-selectivity between Ia and Ib was abolished under these conditions. GST-SNX9 and GST-intersectin I A were standouts since these SH3 domains retained greatest activity in high salt, as also observed for brain dynamin I, and retained the same relative preference for Ib that was observed with low salt. GST-SNX9 SH3 was a significantly better stimulator of Ib in the presence of high salt, while no significant differences were observed between the two salt conditions for Ia (Fig 3A). The effect of GST-intersectin I SH3A was the opposite, showing significantly greater selectivity for Ia in the presence of high salt (Fig 3B). Together these results indicate that the middle domain α2 helix is one of the determining factors of the activity of the G domain in response to SH3-PRD interactions. These overall major effects of the two splice variants on mediating the ability of different GST-SH3 domains to stimulate dynamin activity indicate that the middle domain allosterically regulates the G domain function. Such an allosteric mechanism may help explain the observed disconnect between extent of oligomerisation and activity.

### Effect of GST-SNX9 SH3 on a mixture of dynamin I splice variants

**Fig 4:**
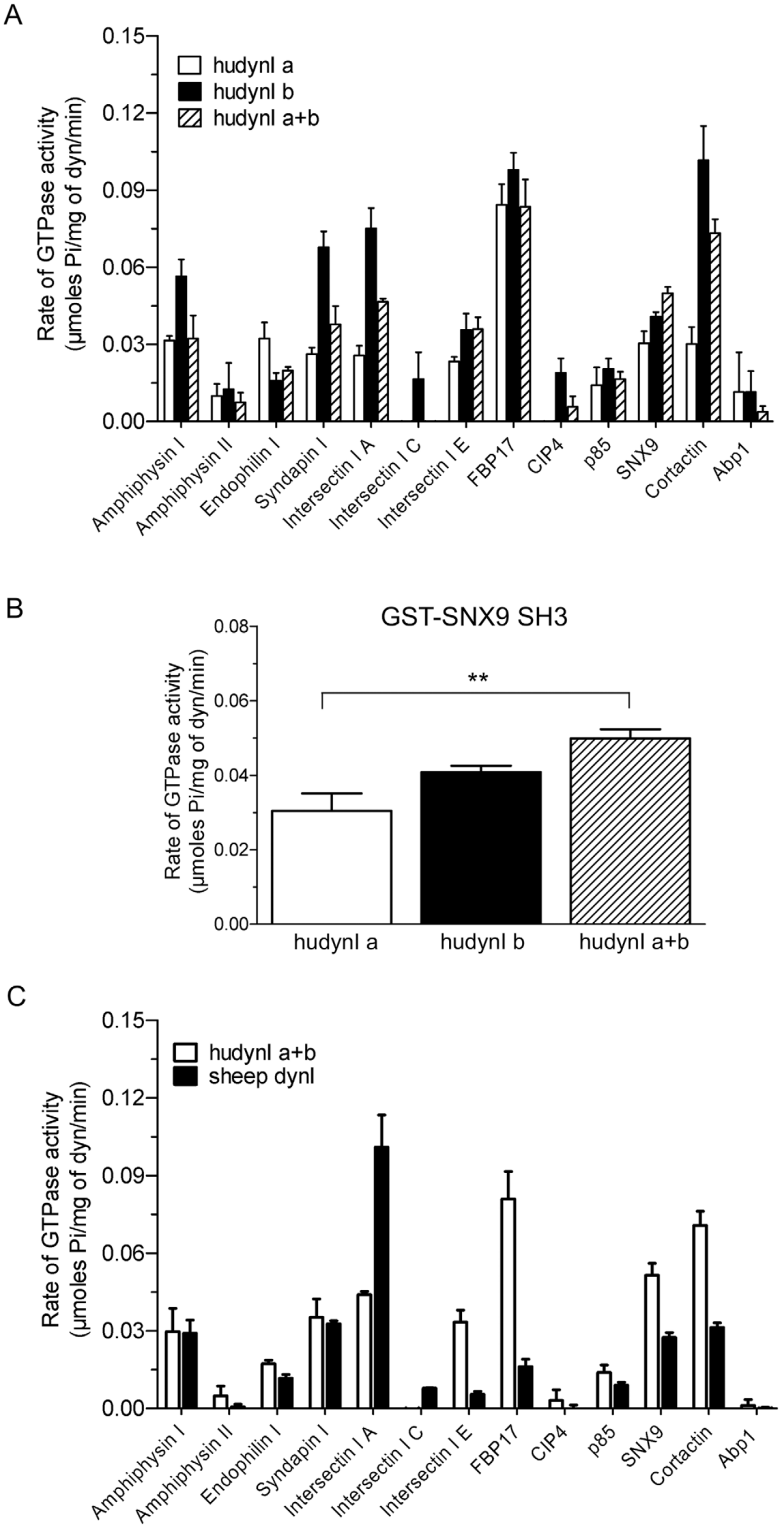
Comparison of the ability of 13 GST-SH3 domains to stimulate the rate of a mix of middle domain splice variants. **(A)** The effect of GST-SH3 domains (40 μg/ml) on the GTPase rate of human dynamin Iab+bb (hudynI a+b) splice variants, which were mixed together at an equal molar ratio to a final concentration of 100 nM / 10 μg/ml. These results are compared to the effect of GST-SH3 domains on the GTPase rate of individual Iab or Ibb splice variants in the presence of low salt (30 mM NaCl) (from Fig 2A). All data is shown as mean ± S.E.M. and n = 3. **(B)** Statistical analysis by one-way ANOVA followed by Bonferroni’s post-test applied to the GST-SNX9 SH3 data for the three human dynamin I samples from (A) (***, p < 0.001). **(C)** The GST-SH3 domain stimulated rate of GTPase activity for combined human dynamin Iab+bb (mixed at a 1:1 molar ratio) from Fig 3A is compared to the effect of GST-SH3 domain on the rate of endogenous purified sheep dynamin I GTPase activity from Fig 1A. The assays were done in the presence of low salt (30 mM NaCl). The data is shown as means ± S.E.M. and n = 3.

Based on the knowledge and that dynamin I and II can form heterodimers *in vitro* [42], we next asked whether mixing the two recombinant human splice variants might alter their SH3 domain response. The Ia and Ib variants were combined in equal amounts (a+b) then stimulated with the GST-SH3 domains in the presence of low salt and compared with activity of single splice variants from data in Fig 2A. Each GST-SH3 domain, except that of SNX9, stimulated the combined dynamin to either the same extent as one of the single splice variants or to an extent that is an average of the two (Fig 4A). Only GST-SNX9 SH3 produced an increased stimulation of the Ia+b mix, especially relative to the ‘a’ single splice form (Fig 4B). This further distinguishes the action of SNX9 SH3 from that of the other SH3 domains in its stimulation of dynamin I GTPase activity.

A species comparison was performed between sheep dynamin I and the human dynamin Ia+b mix of two human dynamin middle domain splice variants (Fig 4C). Human dynamin Ia+b stimulated activity tended to be higher than that of sheep dynamin I, with GST-intersectin I SH3A being a notable exception. The results did not show whether species differences might account for the activity differences, since no discernible pattern could be detected.

## Discussion

We have performed the most extensive parallel analysis to date of isolated SH3 domains and their interaction with dynamin. The SH3-PRD interaction stimulated dynamin GTPase activity and oligomerisation to varying extents across the entire panel of SH3 domains tested. This suggests that SH3-PRD binding acts as a heterotropic allosteric regulator for the G domain and reveals an unexpected role for the PRD in transmitting different assembly signals to the G domain. We identified the middle domain α2 helix as one of the determining factors in the network of allosteric signal transmission, suggesting the PRD and middle domain are part of the same allosteric communication channel. Due to the unique location of the α2 helix between dynamin dimeric subunits this allosteric channel is likely to specifically occur within the dynamin oligomer rather than the dimer.

Dynamin GTPase activity is widely held to be a direct reflection of the extent of assembly [4,5,16] but, surprisingly, this was not observed consistently in our study. If activity is not always correlated with the extent of assembly, it may instead correlate to specific modes of non-helical assembly. Therefore SH3 binding to dynamin may stabilise specific assembled dynamin conformers that result in uniquely assembled oligomers with different GTP hydrolysis rates. The recently described 'morpheein' allosteric mode describes a class of proteins that form different polymeric complexes that are dependent on the unique conformations of the unassembled unit [43]. Upon binding the PRD, the SH3s may stabilise distinct conformations of the dynamin tetramer building block, which both encourages oligomerisation and regulates G domain activity. Under this hypothesis the different hydrolysis rates of dynamin-SH3 complexes represent distinct oligomers with varying GTPase activities. This hypothesis is supported by the observation that GST-intersectin I SH3A forms an unusually compact dynamin ring structure which is very stable and unable to change conformation [29].

Allosteric regulation of the G domain resulting from different PRD-SH3 interactions appears to be mediated by the α2 helix of the middle domain. The identity of this helix is under natural variation through splicing, which we show modulates the activity stimulation induced by SH3 binding. The splicing produces two helices that are each highly sequence-conserved across mammalian species. The α2 helix makes a direct intermolecular contact with the BSE of neighbouring dynamin molecules within the tetramer, forming a ‘dimer-dimer’ interface referred to as interface 5 [2,13]. The BSE itself has also been implicated as a remarkably dynamic domain that appears to respond to the catalytic cycle of the G domain [44,45]. We propose that the BSE may also respond to the binding state of the PRD via the dimer-dimer interface in order to allosterically regulate G domain activity.

The remarkable variety of allosteric effects experienced by the G domain in response to SH3 binding indicates specificity of signalling input into the allosteric network of dynamin. This is potentially made possible because of the large number of SH3 binding motifs within the dynamin I PRD. There are 13 of these motifs (PxxP), at least 5 of which have been shown to differentially bind to SH3 domain proteins [46]. The majority of binding motifs for the SH3 domains used in this study have not been mapped. Of those that have been mapped, only endophilin I SH3 and syndapin I SH3 share a site (Site 2 _786_ **P**AV**P**_789_) [46]. However, endophilin I also binds site 3 (Sites 2 + 3, _786_ **P**AV**PP**AR**P**_793_) preventing further testing of this hypothesis in this study.

The PRD of dynamin is a signalling entry point into the allosteric network of dynamin that regulates distinct assembly modes and differential G domain activity. The assembly and activity properties of dynamin, while often functionally coupled, can therefore be independently regulated. This is evidenced by SH3 domains that are strong stimulators of one property but not the other. The α2 helix of the middle domain is part of this network, splicing of which can modulate the SH3 response of the G domain, potentially through modification of the dimer-dimer interface between the BSE and middle domain. This putative network across 4 domains of dynamin represents the first evidence of direct allosteric communication between regulatory binding proteins and G domain.

## Supporting Information

S1 Fig Relative binding of 13 GST-SH3 domains to dynamin in rat brain synaptosomes. The relative binding of dynamin to individual GST-SH3 domains (~3 μg) was compared by performing pull-down analysis using rat brain synaptosome lysate. The pull-down was resolved on 10% SDS-acrylamide gels and a Coomassie Blue stained gel is shown. The position of dynamin and the synapsin doublet was determined by Western blot and by Mass Spectrometry of cut bands (not shown). The position of each purified GST-SH3 domain is shown. The image is representative of n = 3 independent experiments.
(TIF)

S2 Fig Sedimentation analysis of dynamin assembly by 13 GST-SH3 domains. **(A-C)** Sedimentation analysis showing the effect of 13 GST-SH3 domains (40 μg/ml) on sedimentation of sheep dynamin I (100 nM / 10 μg/ml) under PA conditions in the presence of low salt (30 mM NaCl). Following the incubation of each GST-SH3 with dynamin, filter spin cups were used to collect the oligomerised dynamin (pellet) and supernatant. All the samples were resolved on 10% SDS-acrylamide gels and the protein was visualised using Coomassie Blue. The gel in **(B)** shows dynamin incubated with PS liposome (10 μg/ml) as a maximum assembly control. The images are representative of n = 3 experiments with the samples run in duplicate in each experiment. *Abbreviations*: AmphI/II, amphiphysin I/II; Endo I, endophilin I; ITNA/C/E, intersectin I A/C/E; SndpnI, syndapin I.
(TIF)

S3 Fig Amino acid sequence of the dynamin I middle domain α2 helix splice variants ‘a’ and ‘b’ using CLUSTALW omega. Middle domain splicing of dynamin I (dynI) arises from two alternate exons coding for the residues 400–445 of the middle domain. The differences in residues between sequences are highlighted in red. **(A)** Human dynamin I ‘a’ vs. ‘b’. Species comparison of dynamin I middle domains **(B)** ‘a’ and **(C)** ‘b’. *Abbreviations*: bdynI, bovine (cow); hudynI, human; mdynI, mouse; odynI, ovine (sheep); rdynI, rat.
(TIF)

## Abbreviations

Abp1: Actin binding protein I
AmphI/II: amphiphysin I/II
CIP4: Cdc42 interacting protein 4
Endo I: endophilin I
FBP17: Formin Binding Protein 17
GST: glutathione-*S*-transferase
GTP: Guanosine-5'-triphosphate
hudynI: human dynamin I
ITNA/C/E: intersectin I A/C/E
PRD: proline-rich domain
SH3: Src homology 3
SndpnI: syndapin I
SNX9: sorting nexin 9
