# Supplementary figures and images for: SH3 Domains Differentially Stimulate Distinct Dynamin I Assembly Modes and G Domain Activity
Source: PLoS One. 2015 Dec 10;10(12):e0144609. doi: 10.1371/journal.pone.0144609 (PMC4687643; doi:10.1371/journal.pone.0144609)

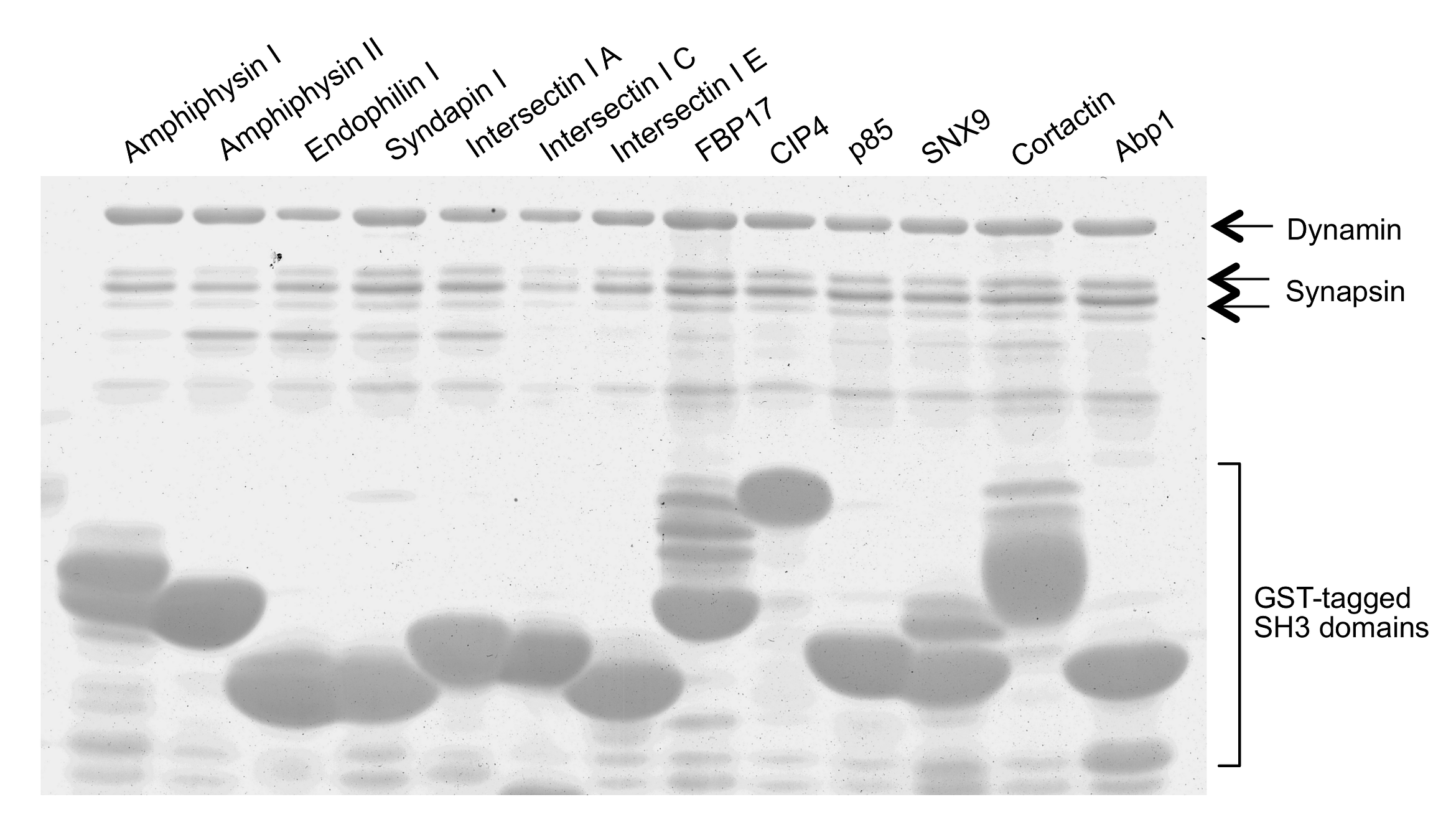

Supplement: S1 Fig — The relative binding of dynamin to individual GST-SH3 domains (~3 μg) was compared by performing pull-down analysis using rat brain synaptosome lysate. The pull-down was resolved on 10% SDS-acrylamide gels and a Coomassie Blue stained gel is shown. The position of dynamin and the synapsin doublet was determined by Western blot and by Mass Spectrometry of cut bands (not shown). The position of each purified GST-SH3 domain is shown. The image is representative of n = 3 independent experiments. (TIF) [file pone.0144609.s001.tif]

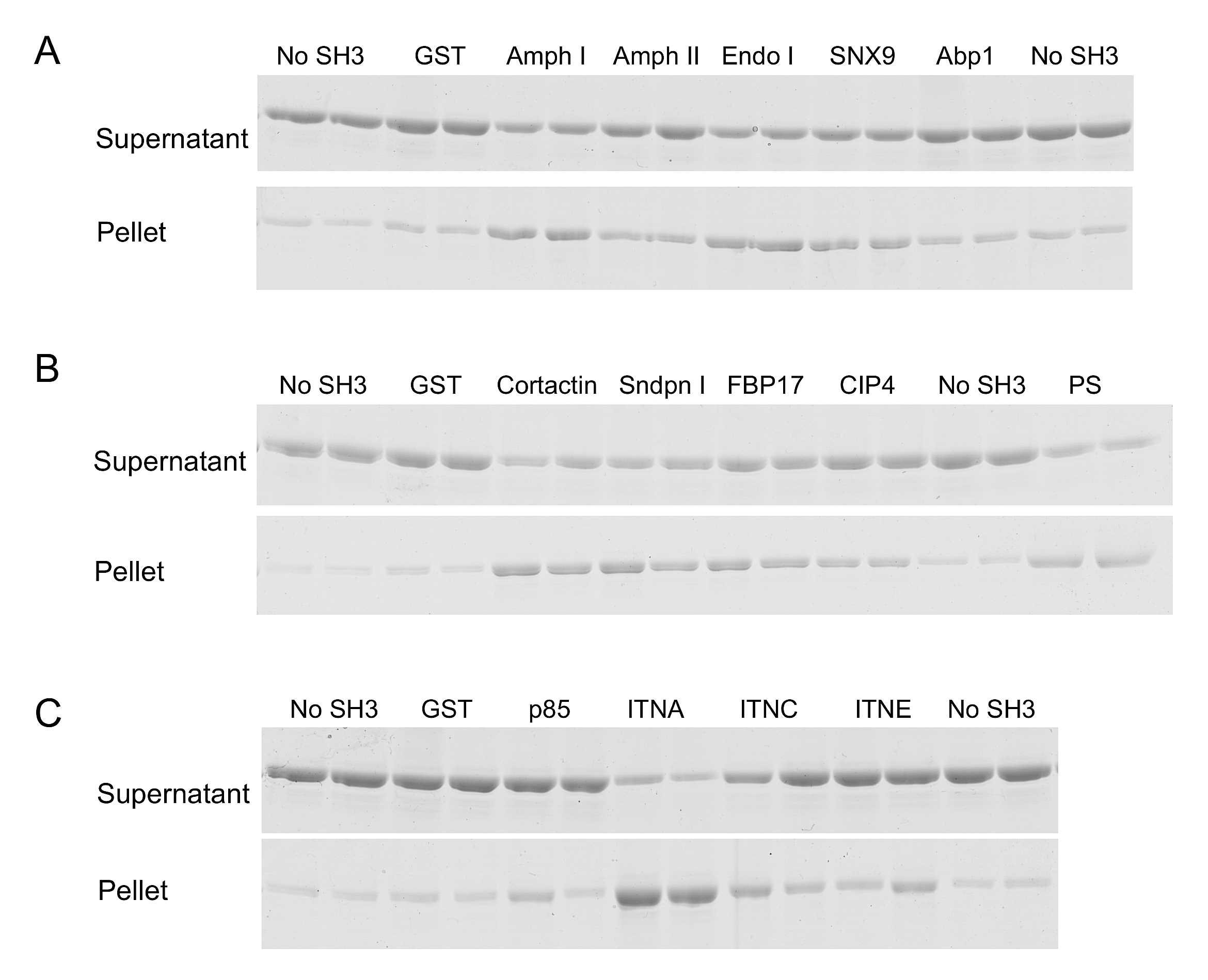

Supplement: S2 Fig — (A-C) Sedimentation analysis showing the effect of 13 GST-SH3 domains (40 μg/ml) on sedimentation of sheep dynamin I (100 nM / 10 μg/ml) under PA conditions in the presence of low salt (30 mM NaCl). Following the incubation of each GST-SH3 with dynamin, filter spin cups were used to collect the oligomerised dynamin (pellet) and supernatant. All the samples were resolved on 10% SDS-acrylamide gels and the protein was visualised using Coomassie Blue. The gel in (B) shows dynamin incubated with PS liposome (10 μg/ml) as a maximum assembly control. The images are representative of n = 3 experiments with the samples run in duplicate in each experiment. Abbreviations: AmphI/II, amphiphysin I/II; Endo I, endophilin I; ITNA/C/E, intersectin I A/C/E; SndpnI, syndapin I. (TIF) [file pone.0144609.s002.tif]
